# Discovering the mechanics of ultra-low density elastomeric foams in elite-level racing shoes

**DOI:** 10.1007/s00366-026-02398-y

**Published:** 2026-09-10

**Authors:** Jeremy A. McCulloch, Scott L. Delp, Ellen Kuhl

**Affiliations:** Departments of Mechanical Engineering and Bioengineering, Stanford University, 318 Campus Drive, Stanford, CA 94305 USA

**Keywords:** Hyperelasticity, Mechanical testing, Constitutive modeling, Elastomeric foam, Polymeric foam, Automated model discovery

## Abstract

Ultra-low-density elastomeric foams enable lightweight systems that combine high compliance with efficient energy return. Their mechanical response is inherently complex, characterized by high compressibility, nonlinear elasticity, and microstructural heterogeneity. In high-performance racing shoes, these foams are critical for low weight, high cushioning, and efficient energy return; yet, their constitutive behavior remains difficult to model and poorly understood. Here we integrate mechanical testing and machine learning to discover the mechanics of two ultra-low density elastomeric polymeric foams used in elite-level racing shoes. Across uniaxial tension, confined and unconfined compression, and simple shear, both foams exhibit pronounced tension–compression asymmetry, negligible lateral deformation consistent with an effective Poisson’s ratio close to zero, and low hysteresis indicative of an efficient energy return. Quantitatively, at a strain rate of 0.25/s, both foams provide a similar compressive stiffness (E = 268±16 kPa vs. E = 299±29 kPa), while one foam exhibits a 42% higher tensile stiffness (E = 884±69 kPa vs. E = 623±96 kPa), and nearly double the shear stiffness (G = 219±20 kPa vs. G = 117±24 kPa), implying a substantially greater lateral stiffness at a comparable vertical energy return (83.3±1.5% vs. 88.9±1.8%). By integrating these data into constitutive neural networks, paired with sparse regression, we discover compact, interpretable single-invariant models–supplemented by mixed-invariant or principal-stretch based terms–that capture the unique signature of the foams with ${\textsf {R}}^{\textsf {2}}$ values close to one across all loading modes. From a human performance perspective, these models have the potential to improve gait-level simulations with high-performance racing shoes to quantify running economy, performance enhancements, and injury risks on an individual athlete level. More broadly, this work establishes a scalable and interpretable approach for constitutive modeling of highly compressible, ultra-light elastomeric foams with applications to wearable technologies, soft robotics, and energy-efficient mobility systems. Source code, data, and examples are available at https://github.com/LivingMatterLab/CANN.

## Introduction

The two-hour marathon barrier remains a defining frontier of human performance. The current world record of 2:00:35 was set at the 2023 Chicago Marathon by Kelvin Kiptum wearing a prototype of the Nike Alphafly 3, a high-performance racing shoe with a carbon-fiber plate embedded in an ultra-low density elastomeric foam [1]. Over the past decade, the introduction of racing shoes that combine carbon-fiber plates and ultra-light elastomeric foams has been associated with substantial performance improvements among both recreational and elite athletes, particularly in long-distance running [2]. Compared to earlier racing shoes, carbon-fiber-plated footwear exhibits a reduced weight, increased cushioning, and high energy return, features that collectively improve running economy by altering lower-limb mechanics and reducing energy dissipation [3].

### Increasing the performance of next-generation racing shoes.

Extensive studies of the first prominent carbon-fiber-plated shoe, the Nike Vapor-fly, suggest that it can reduce the metabolic cost of running at a fixed speed by up to 4% [4], or, equivalently, increase the speed by 4% at constant metabolic demand [5]. Inspired by these early success stories, various brands are now producing their own versions of carbon-fiber-plated shoes in an attempt to increase speed and decrease metabolic cost [6]. A recent comparison between seven carbon-fiber-plated shoes and one conventional shoe revealed that the Nike Vaporfly 2, the Nike Alphafly, and the Asics Metaspeed Sky achieved the greatest reduction in metabolic demand [7]. The study concluded that the longitudinal bending stiffness played a minor role in metabolic savings, and that, with all shoe weights nearly equal, the greatest differentiating factor was the midsole foam. Increasing evidence suggests that the midsole foam–rather than the carbon-fiber plate–may hold the key to breaking the two-hour marathon barrier [8].

### Reducing injury risk by balancing cushioning and stability.

While the major focus of carbon-fiber-plated racing shoes lies in increasing running performance, it is equally important to understand whether these shoes might increase the prevalence of running-related injuries. Some studies have proposed that more energy is stored in the shoe and less is stored in muscles and tendons, which may decrease the risk of running injuries [3]. However, a recent case study of six stress fractures–sustained while wearing carbon-fiber-plated shoes–suggests that these shoes increase displacements of the navicular and cuneiform bones and modify forces to the hindfoot, which could increase injury risk [9]. By design, carbon-fiber–plated racing shoes are primarily optimized for straight-line running. The ultra-low density foams of their midsole exhibit an inherently low shear stiffness as a consequence of cellular solids scaling, meaning the shear modulus decreases super-linearly with decreasing density [10]. This low shear stiffness reduces stability in cornering and uneven terrain, and increases injury risk.

### Constitutive accuracy as the missing link in gait simulation.

Computational models of gait provide valuable guidance how to increase performance or reduce injury risk. The ground reaction forces and the joint angles of the hip, knee, and ankle are straightforward to measure, and can be integrated into a generative machine learning model [11]; however, this model and many others neglect the mechanics of the foot and the shoe. Current attempts to model foot and shoe take magnetic resonance images [12], segment the image stack, assign constitutive models to the different materials, and impose boundary conditions [13]. In this process, the major source of error is the selection of constitutive models for the foot and the shoe. This challenge is reflected in a study, which models the foot and the shoe using a six-degree of freedom model: The model accurately reconstructs joint kinematics, but predicts inaccurate ground reaction forces, and its results vary greatly for small variations in running terrain [14]. Thus, further improvements in both biomechanical modeling of the foot [15] and constitutive modeling of the shoe [16] are critically needed to improve gait performance and injury models.

### Mechanics of ultra-low density elastomeric foams.

To efficiently store mechanical energy during the stance phase and return it during toe-off, modern high-performance racing shoes employ ultra-low-density elastomeric foams. Elastomers can undergo large, reversible deformations that enable substantial elastic energy storage, while maintaining mechanical robustness under repeated loading [17]. Their porous microstructure reduces mass and increases compliance, both of which are critical in endurance running. Under uniaxial compression, elastomeric foams exhibit a characteristic nonlinear stress–stretch response: an initial linear elastic regime associated with reversible cell wall bending; a plateau regime associated with cell wall buckling; and a densification regime associated with pore collapse and rapidly increasing stiffness [18]. This S-shaped mechanical response enables a superior energy return at relatively low stresses when deformations remain below the densification regime. At the same time, peak performance benefits of modern high-performance racing shoes are typically limited to 150–300 miles of use [8]. While carbon-fiber plates exhibit minimal structural degradation, the ultra-low density polymeric foam undergoes viscoelastic fatigue under cyclic loading, leading to a progressive loss of resilience and energy return [19]. It is becoming increasingly clear that this limited lifespan is governed by midsole foam mechanics, rather than carbon-fiber plate integrity: Designing the midsole foam is a trade off between performance, stability, and durability.

### Dual-foam midsole architectures.

Not surprisingly, the mechanical properties of ultra-low-density polymeric foams are closely guarded and largely proprietary. Design laboratories fine-tune midsole foams by modulating polymer chemistry, void fraction, and foam microstructure, including pore size, pore shape, and the presence of microfillers [19]. As an illustrative example, Asics employs two proprietary midsole foams in its top-of-the-line racing shoe, FF LEAP$^\mathsf{{TM}}$ and FF TURBO$^\mathsf{{TM}}$ PLUS. Notably, Asics is the only major manufacturer that offers two variants of its racing shoe tailored to different running styles: the Metaspeed Edge, optimized for cadence, and the Metaspeed Sky, optimized for stride-length [7]. The primary distinction between these designs is the vertical arrangement of the two foams: In the Edge, FF LEAP$^\mathsf{{TM}}$ sits above the carbon-fiber plate and FF TURBO$^\mathsf{{TM}}$ PLUS below, while in the Sky, this configuration is reversed. However, because the mechanical properties of these foams are not publicly disclosed, it remains unclear to which extent this dual-foam arrangement truly provides a functional mechanical advantage.

### Automated model discovery.

Ultra-lightweight polymeric foams have undergone extensive experimental and theoretical study over several decades, resulting in a broad range of constitutive models that span cellular solids theory, hyperelasticity, and viscoelasticity [10]. Despite this progress, the selection of the appropriate foam models remains based on user experience and personal preference, rather than objective selection criteria. Constitutive artificial neural networks enable the automated discovery of constitutive models, while simultaneously identifying optimal model parameters [20]. This deep learning framework identifies hyperelastic constitutive models for isotropic [21], transversely isotropic [22], and orthotropic [23] materials, and extends naturally to viscoelastic [24] and inelastic material behavior [25]. These approaches apply across a wide range of materials, including biological tissues [26], foods [27], and synthetic materials [28]. While most existing studies focus on incompressible materials, recent work has introduced a bulk stiffness contributions into the constitutive neural network [29]. Other machine-learning approaches also address the constitutive behavior of elastomeric foams [30]. However, these vanilla type neural networks omit physics-based constraints in both the network architecture and the loss function and fail to leverage the extensive theoretical foundation of continuum mechanics and constitutive modeling. Here we use constitutive neural networks to discover physic-based models for ultra-low density foams used in high-performance racing shoes.

## Methods

In this study, we characterize two ultra-low density elastomeric polymeric foams the FF TURBO$^\mathsf{{TM}}$ PLUS and FF LEAP$^\mathsf{{TM}}$ used in the Asics Metaspeed carbon-fiber-plated racing shoes. We test both foams in uniaxial tension, unconfined and confined compression, and simple shear, and use the experimental data to discover hyperelastic constitutive models for the foams using a customized isotropic constitutive neural network.

**Fig. 1:**
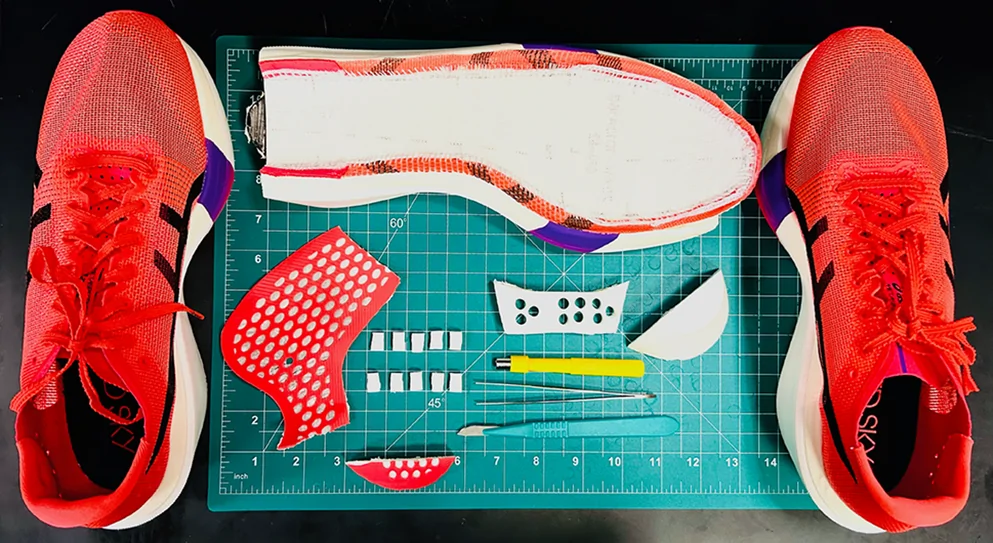
**Sample preparation.** We prepare samples from the ASICS Metaspeed Sky and Edge racing shoes by removing the rubber outsole and separating the carbon-fiber plate from the underlying foam. We section the extracted foam into slabs, and cut rectangular samples for uniaxial tension testing and cylindrical samples for unconfined and confined compression, and shear testing. The images illustrate shoe disassembly, foam sectioning, and specimen fabrication

**Fig. 2:**
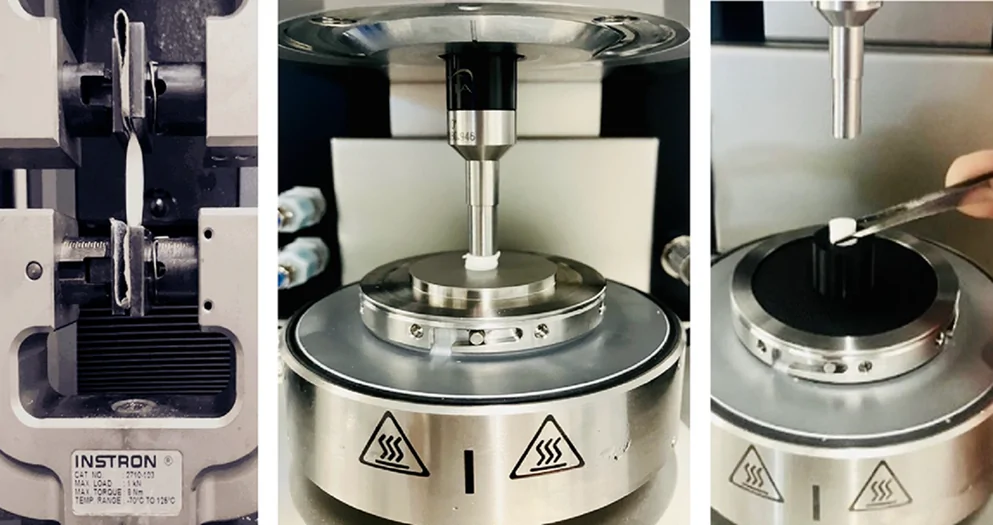
**Mechanical testing.** We test five samples of each foam in uniaxial tension, left, unconfined compression, middle, and confined compression, right

### Sample preparation

We extract samples from Asics Metaspeed Sky and Edge racing shoes. To start, we remove the rubber outer sole from each shoe by cutting with a surgical blade and gently pulling on the rubber. Then, we use a hot wire foam cutter (YaeTek Micromot Hot Wire ThermoCut Foam Cutting Machine, Yaemart, Duluth, GA) to cut along the bottom side of the carbon-fiber plate and separate it from the foam below. We then cut this piece of foam into 10 mm slabs along the length of the shoe. For the uniaxial tension tests, we cut rectangular samples of 5 mm thickness, 10 mm width, and 50 mm length. For the compression, shear, and confined compression tests, we create cylindrical samples of 8 mm diameter and 10 mm height using a biopsy punch. Before testing, we measure the exact dimensions of each samples using calipers. Figure 1 shows details of the sample preparation.

### Mechanical testing

We test *n=5* samples of the FF LEAP$^\mathsf{{TM}}$ and *n=5* samples of the FF TURBO$^\mathsf{{TM}}$ PLUS in uniaxial tension, unconfined and confined compression, and simple shear. Since the foams experience fast loading during running, we choose the fastest possible loading rate at which there are no significant inertial artifacts present in the data. Figure 2 shows the experimental test setups for uniaxial tension, unconfined compression and simple shear, and confined compression, from left to right.

#### Uniaxial tension.

For the uniaxial tension tests, we use an Instron 5848 (Instron, Canton, MA) equipped with a 100 N load cell. We tightly clamp 15 mm length at each end of the sample using grips with sandpaper, to leave a gauge length of approximately 20 mm. We zero the load cell to the weight of the foam sample, and then apply a pre-strain of 0.1, corresponding to a pre-load of approximately 2 kN, by gradually increasing the deformation until the axial force stabilizes above 0.1 N. After preloading, we cyclically load the sample to a maximum stretch of *λ = 1.3* at a rate of $\dot{\lambda } = 0.25$/s for six cycles.

#### Unconfined and confined compression.

For the unconfined compression tests, we use a HR20 discovery hybrid rheometer (TA Instruments, New Castle, DE) equipped with a 10 N load cell and an 8 mm tool. For this test, the cylindrical sample only contacts the device on its top and bottom surfaces is free to expand in the lateral direction. We again apply a pre-strain of 0.1, corresponding to a pre-load of approximately 2 kN, by lowering the end effector 50 *μ *m at a time until the axial force stabilizes above 0.1 N. After preloading, we cyclically load the sample to a minimum stretch of *λ = 0.4* at a rate of $\dot{\lambda } = 0.25$/s for six cycles; and then compress the sample to the maximum force of the load cell of approximately 9 N at the same strain rate. For the confined compression tests, we mount the cylindrical sample into a custom base plate with an 8 mm inner diameter confined compression chamber, and following the same testing protocol. For this test, the cylindrical sample contacts the device on all sides and cannot expand in the lateral direction.

#### Simple shear.

For the shear tests, we use a HR20 discovery hybrid rheometer (TA Instruments, New Castle, DE) equipped with a custom 3D printed end effector and a platform with sandpaper glued to both. The sandpaper minimizes slippage between the sample and the device without using of an adhesive, which may alter the foam properties. First, we apply a preload similar to the case of unconfined compression. Then, we compress the samples to a stretch of *λ = 0.8* at a rate of $\dot{\lambda } = 0.25$/s. Then, we apply a sinusoidal angular displacement with a maximum shear strain of *γ = 0.25* and a maximum shear strain rate of $\dot{\gamma } = 0.25$/s, which corresponds to an angular frequency of 1 rad/s.

### Data processing

For each of the four mechanical testing modes in Sect. 2.2, for each foam type, we aim to derive a single stress-stretch curve, which represents the average hyperelastic response of that foam. We convert the raw load–displacement recordings into stretch-stress measurements, average the loading and unloading curves for each sample, and then average across all *n=5* samples. The the following subsections outline the precise process for each loading mode.

#### Uniaxial tension.

For each sample, we measure the initial cross sectional area *A* and the initial length *L* between the clamps. Throughout the experiment, we record the axial force *F*(*t*) and the displacement *u*(*t*) over time *t*. We convert both measurements into the axial stretch *λ * and the Piola stress $P_{{\text {11}}}$,

1 $$\begin{aligned} \lambda = 1 + \frac{u}{L} \quad \text{ and } \quad P_{\text {11}} = \frac{F}{A}\,. \end{aligned}$$

By visually observing the tension experiment and inspecting the stress-stretch curve, we conclude that a part of the behavior is inelastic, since the stress reaches zero during unloading at stretches significantly larger than one. For all further analyses, we only consider the stretch range of $1.0 \le \lambda \le 1.3$. To obtain the stretch-stress curves $P_{\text {11}}(\lambda )$ for each sample, we exclude the first loading and unloading curves, average the remaining ten curves, and zero the stresses such that $P_{\text {11}}|_{\lambda =1} = 0$. From the *n=5* individual curves, we compute the mean $P_{\text {11}}(\lambda )$ and the standard deviation $\sigma _{P_{\text {11}}}$ across all five samples.

#### Unconfined and confined compression.

For each sample, we measure the initial cross sectional area *A* and the initial sample height *H*. Throughout the experiment, we record the axial force *F*(*t*) and the deformed height of the sample *h*(*t*) over time *t*. We convert both measurements into the axial stretch *λ * and the Piola stress $P_{\text {11}}$,

2 $$\begin{aligned} \lambda = \frac{h}{H} \quad \text{ and } \quad P_{\text {11}} = \frac{F}{A} \,. \end{aligned}$$

For all further analyses, we consider the stretch range of $0.4 \le \lambda \le 1.0$. For each sample, we exclude the first loading and unloading curves, average the remaining ten curves, and zero the stresses such that $P_{\text {11}}|_{\lambda =1} = 0$. From the *n=5* individual curves, we compute the mean $P_{\text {11}}(\lambda )$ and the standard deviation $\sigma _{P_{\text {11}}}$ across all five samples. We use the same procedure for both unconfined and confined compression.

#### Simple shear.

For each sample, we measure the initial radius *R* and the initial sample height *H*. Throughout the experiment, we record the torsion angle *ϕ (t)* and the torque *T*(*t*) over time *t*. We convert both measurements into the shear strain *γ * and the Piola stress $P_{\text {12}}$,

3 $$\begin{aligned} \gamma = \frac{R}{H} \, \phi \quad \text{ and } \quad {P}_{\text {12}} = \frac{2}{\pi R^3} \, T \,, \end{aligned}$$

where we calculate the torque *T* by integrating the shear stress $P_{\text {12}}$ times its moment arm *r* across the cross section, ${\text {d}}A = r \, {\text {d}}r \, {\text {d}} \theta$, of the sample, $T = 2\pi \int _{r=0}^{R} P_{\text {12}} \,r^2 \, {\text {d}}r$, assume the following explicit shear stress–strain relation, $P_{\text {12}} = 2\, [\, \partial \psi / \partial I_1 + \partial \psi / \partial I_2 \,] \, \gamma$, and apply the trapezoidal rule, $\int _{r=0}^R f(r) {\text {d}}r \approx R \, [f(0) + f(R)]/2$, to numerically approximate the integral [31]. These equations are exact for linear materials, but they may very well under- or over-estimate the shear stress ${P}_{\text {12}}$ for nonlinear hyperelastic materials depending on whether the shear stress-stretch curve is concave or convex. For all further analyses, we consider shear stretches, $\gamma \le 0.2$, for which the shear response is close to linear. From the *n=5* individual curves, we compute the mean $P_{\text {12}}(\gamma )$ and the standard deviation $\sigma _{P_{\text {12}}}$ across all five samples.

#### Linear elastic stiffness and energy return.

Using the stress-stretch pairs of the tension, compression, and shear experiments in Figs. 4 and 5, for relative deformations up to *10%,* we perform a linear regression to estimate the linear elastic stiffness in tension ${\textsf {E}}_{\text {ten}} = \boldsymbol{\epsilon }_{\text {ten}} \cdot \boldsymbol{\sigma }_{\text {ten}} / \boldsymbol{\epsilon }_{\text {ten}} \cdot \boldsymbol{\epsilon }_{\text {ten}},$ compression ${\textsf {E}}_{\text {com}} = \boldsymbol{\epsilon }_{\text {com}} \cdot \boldsymbol{\sigma }_{\text {com}} / \boldsymbol{\epsilon }_{\text {com}} \cdot \boldsymbol{\epsilon }_{\text {com}},$ and shear ${\textsf {G}}_{\text {shr}} = \boldsymbol{\gamma }_{\text {shr}} \cdot \boldsymbol{\tau }_{\text {shr}} / \boldsymbol{\gamma }_{\text {shr}} \cdot \boldsymbol{\gamma }_{\text {shr}},$ where $\boldsymbol{\epsilon }_{\text {ten}}, \boldsymbol{\epsilon }_{\text {com}}, \boldsymbol{\gamma }_{\text {shr}}$ are vectors that contain the discrete strain values up to *10%* across all five foams and $\boldsymbol{\sigma }_{\text {ten}}, \boldsymbol{\sigma }_{\text {com}}, \boldsymbol{\tau }_{\text {shr}}$ are vectors of the associated stresses. Using the full loading and unloading curves of the tension, compression, and shear experiments in Figs. 4 and 5, across the entire range of deformation, we calculate the relative energy return, $\eta = {\textsf {E}}_{\text {unload}} / {\textsf {E}}_{\text {load}},$ as the ratio between the areas under the unloading curve ${\textsf {E}}_{\text {unload}}$ and loading curve ${\textsf {E}}_{\text {load}}.$ We report the stiffnesses, ${\textsf {E}}_{\text {ten}}, {\textsf {E}}_{\text {com}},{\textsf {E}}_{\text {shr}},$ and the relativ energy returns, $\eta _{\text {ten}},\eta _{\text {com}},\eta _{\text {shr}},$ for both foams as means ± standard deviations.

### Continuum mechanics

#### Kinematics.

When comparing the stress-stretch curves from the unconfined and confined compression tests, we do not observe significant differences. We conclude that the effective Poisson’s ratio of both foams is approximately zero, which we confirm by visual inspection of the sample during unconfined compression. Therefore, we assume that in uniaxial tension and compression, the transverse stretch is one. With the additional assumption of a homogeneous deformation field, the deformation gradient becomes

4 $$\begin{aligned} \boldsymbol{F} = \left[ \, \begin{array}{ccc} \lambda & \gamma & 0 \\ 0 & \alpha & 0\\ 0 & 0 & 1 \end{array} \, \right], \end{aligned}$$

where *λ * is the axial stretch, *α * is the transverse stretch, and *γ * is the shear strain. Next, we compute the right Cauchy Green deformation tensor,

5 $$\begin{aligned} \boldsymbol{C} = \left[ \, \begin{array}{ccc} \lambda ^2 & \lambda \gamma & 0 \\ \lambda \gamma & \alpha ^2 + \gamma ^2 & 0\\ 0 & 0 & 1 \end{array} \, \right]. \end{aligned}$$

#### Invariant-based strain energy.

We introduce the set of invariants,

6 $$\begin{aligned} \begin{array}{lcl} I_1 & =& {{\,\textrm{tr}\,}}(\boldsymbol{C}) = 1 + \alpha ^2 + \lambda ^2 + \gamma ^2 \\[4.pt] I_2 & =& \frac{1}{2}[\, I_1^2 - \boldsymbol{C}:\boldsymbol{C} \,] = \alpha ^2 + \gamma ^2 + \lambda ^2 + \alpha ^2\lambda ^2 \\[4.pt] J & =& \det (\boldsymbol{F}) = \lambda \, \alpha \,. \end{array} \end{aligned}$$

and assume a strain energy function for which the contributions of these three invariants are fully decoupled,

7 $$\begin{aligned} \psi (I_1, I_2, J) = \psi _{I_1}(I_1) + \psi _{I_2}(I_2) + \psi _{J}(J) \,. \end{aligned}$$

With the partial derivatives of the invariants (6) with respect to *λ,* *γ,* *α, * at *α = 1,*

8 $$\begin{aligned}\begin{array}{lcllcllcl} \displaystyle {\frac{\partial I_1}{\partial \lambda }} & =& 2 \lambda & \displaystyle {\frac{\partial I_1}{\partial \gamma }} & =& 2 \gamma & \displaystyle {\frac{\partial I_1}{\partial \alpha }} & =& 2 \\[8.pt] \displaystyle {\frac{\partial I_2}{\partial \lambda }} & =& 4 \lambda & \displaystyle {\frac{\partial I_1}{\partial \gamma }} & =& 2 \gamma & \displaystyle {\frac{\partial I_1}{\partial \alpha }} & =& 2 + 2\lambda ^2 \\[8.pt] \displaystyle {\frac{\partial J}{\partial \lambda }} & =& 1 & \displaystyle {\frac{\partial I_1}{\partial \gamma }} & =& 0 & \displaystyle {\frac{\partial I_1}{\partial \alpha }} & =& \lambda \,, \end{array} \end{aligned}$$

we compute the components of the Piola stress,

9 $$\begin{aligned} \begin{array}{lclclcl} P_{11} & =& \displaystyle {\frac{\partial \psi }{\partial F_{11}}} & =& \displaystyle {2\lambda \frac{\partial \psi }{\partial I_1} + 4\lambda \frac{\partial \psi }{\partial I_2} + \frac{\partial \psi }{\partial J}} \\[8.pt] P_{12} & =& \displaystyle {\frac{\partial \psi }{\partial F_{12}}} & =& \displaystyle {2\gamma \frac{\partial \psi }{\partial I_1} + 2\gamma \frac{\partial \psi }{\partial I_2}} \\[8.pt] P_{22} & =& \displaystyle {\frac{\partial \psi }{\partial F_{22}}} & =& \displaystyle {2\frac{\partial \psi }{\partial I_1} + [2 + 2\lambda ^2]\frac{\partial \psi }{\partial I_2} + \lambda \frac{\partial \psi }{\partial J}}. \end{array} \end{aligned}$$

To ensure that the stresses vanish in the reference configuration, for *λ = 1,* *γ = 0,* *α = ,1* we need to enforce that $P_{11}|_{\boldsymbol{F}=\boldsymbol{I}} = 0,$ thus

10 $$\begin{aligned} 2 \left. \frac{\partial \psi }{\partial I_1}\right|_{\boldsymbol{F}=\boldsymbol{I}} + 4 \left.\frac{\partial \psi }{\partial I_2}\right|_{\boldsymbol{F}=\boldsymbol{I}} + \left. \frac{\partial \psi }{\partial J}\right|_{\boldsymbol{F}=\boldsymbol{I}} = 0 \,, \end{aligned}$$

while $P_{12}|_{\boldsymbol{F}=\boldsymbol{I}} = 0$ alway holds. For the free energy function in Eq. (7), the above requirement reduces to the overly restrictive condition, ${\partial \psi }/{\partial I_1}|_{I_1=3} = {\partial \psi }/{\partial I_2}|_{I_1=3} = {\partial \psi }/{\partial J}|_{J=1} = 0.$

#### Isochoric-invariant-based strain energy.

Instead of using the set of invariants (6), we introduce the set of isochoric invariants,

11 $$\begin{aligned} \bar{I_1} =\frac{I_1}{J^{2/3}} \, \text{ and } \, \bar{I_2} = \frac{I_2}{J^{4/3}} \, \text{ and } \, J = \text {det} (\boldsymbol{F})\,, \end{aligned}$$

and assume a free energy function,

12 $$\begin{aligned} \psi (\bar{I}_1, \bar{I}_2, J) = \psi _{\bar{I}_1}(\bar{I_1}) + \psi _{\bar{I}_2}(\bar{I_2}) + \psi _{J} (J) \,. \end{aligned}$$

Now, with ${\partial \psi _{\bar{I}_1}}/{\partial \bar{I}_1}|_{\bar{I}_1=3}=0$ and ${\partial \psi _{\bar{I}_2}}/{\partial \bar{I}_2}|_{\bar{I}_2=3}=0$ and $-2{\partial \psi _{\bar{I}_1}}/{\partial \bar{I}_1}|_{\bar{I}_1=3} -4{\partial \psi _{\bar{I}_2}}/{\partial \bar{I}_2}|_{\bar{I}_2=3} + {\partial \psi _{J}}/{\partial J}|_{J=1}=0,$ the zero stress condition in Eq. (10) reduces to

13 $$\begin{aligned} \left. \frac{\partial \psi _{J}}{\partial J}\right|_{J=1} = 0 \,, \end{aligned}$$

which puts no restrictions on the forms of $\psi _{\bar{I}_1}$ and $\psi _{\bar{I}_2}.$ We also require that, in the limits of *J→ 0* or $J\rightarrow \infty,$ the energy becomes infinite, $\psi \rightarrow \infty,$ thus

14 $$\begin{aligned} \lim _{J\rightarrow 0}\partial \psi _{J}(J) = \lim _{J\rightarrow +\infty }\partial \psi _{J}(J) = +\infty \,. \end{aligned}$$

#### Principal-stretch-based strain energy.

It can be useful to express some or all of the terms in the strain energy function in terms of the principal stretches $\{\,\lambda _1,\lambda _2,\lambda _3\,\}$ [32], which are singular values of the deformation gradient $\boldsymbol{F}.$ Here we introduce a principal-stretch-based strain energy [33],

15 $$\begin{aligned} \psi = f(\lambda _1)+f(\lambda _2)+f(\lambda _3) = \sum _{i = 1}^3 f(\lambda _i) \,, \end{aligned}$$

which is isotropic and objective as $\boldsymbol{F} \cdot \boldsymbol{Q},$ $\boldsymbol{Q} \cdot \boldsymbol{F},$ and $\boldsymbol{F}$ all have the same singular values for any proper orthogonal tensor $\boldsymbol{Q}.$ We also require that for no deformation, $\boldsymbol{F} = \boldsymbol{I},$ the strain energy is zero, $\psi |_{\boldsymbol{F}=\boldsymbol{I}} = 3\,f|_{\lambda _i=1} = 0,$ and the stress is zero, so $P_{\text {11}}|_{\boldsymbol{F}=\boldsymbol{I}} = f'|_{\lambda _i=1} = 0,$ thus $f|_{\lambda _i=1} = f'|_{\lambda _i=1} = 0.$ For the uniaxial tension or compression experiments, with *γ = 0,* the principal stretches are $\lambda _1 = \lambda$ and $\lambda _2 = \alpha$ and $\lambda _3 = 1,$ the strain energy is $\psi = f(\lambda ) + f(\alpha ),$ and the normal stresses are

16 $$\begin{aligned} P_{\text {11}} = \frac{\partial \psi }{\partial \lambda } = f'(\lambda ) \, \text{ and } \, P_{\text {22}} = \frac{\partial \psi }{\partial \alpha } = f'(\alpha ) \,. \end{aligned}$$

In our case, with *α = 1,* $P_{22} = f'(1) = 0.$ For the shear experiments, with *α = 1,* the principal stretches are $\lambda _{1,2}^2 = \frac{1}{2}\,[1 + \lambda ^2 + \gamma ^2]$ $\pm \frac{1}{2}\,\sqrt{[ 1 + \lambda ^2 + \gamma ^2]^2 - 4\, \lambda ^2},$ their derivatives with respect to the shear strain *γ * are $\partial \lambda _{1,2}/{\partial \gamma } = \frac{1}{2}\, \gamma [\,1\pm [1 + \lambda ^2 + \gamma ^2]$ $/\sqrt{[1 + \lambda ^2 + \gamma ^2]^2 - 4\lambda ^2} \,] / \lambda _{1,2},$ and the shear stress is

17 $$\begin{aligned} P_{12} =\frac{\partial \psi }{\partial \gamma } = f'(\lambda _1)\frac{\partial \lambda _1}{\partial \gamma } + f'(\lambda _2)\frac{\partial \lambda _2}{\partial \gamma } \,. \end{aligned}$$

### Automated model discovery

To discover the best constitutive model and parameters for ultra-low density elastomeric foams, we create a constitutive neural network which takes the invariants $\{ \bar{I}_1, \bar{I}_2, J \},$ and the principal stretches $\{ \lambda _1, \lambda _2, \lambda _3 \}$ as inputs and outputs the strain energy function *ψ * from which we derive the stress. Specifically, we integrate single-invariant terms, mixed-invariant terms, and principal-stretch terms in a single free energy function of the following form,

18 $$\begin{aligned} \psi = \psi _{\bar{I}_1} + \psi _{\bar{I}_2} + \psi _{J} + \psi _{\bar{I}_1,J} + \psi _{\bar{I}_2,J} + \psi _{\lambda _i}. \end{aligned}$$

In the following, we briefly motivate and introduce these six terms.

#### Single-invariant terms.

Previous work has shown how to build a constitutive neural network for isotropic incompressible materials with the invariants $\{ {I}_1, {I}_2 \}$ as input [20]. Here, we use a similar architecture with linear, quadratic, linear exponential, and quadratic exponential activation functions, but now in terms of the invariant $\bar{I}_1,$

19 $$\begin{aligned} \begin{array}{lclclcl} \psi _{{\bar{I}_{1} }} & = & w_{1} [\bar{I}_{1} - 3] &+& w_{2} [\exp (w_{2}^{*} [\bar{I}_{1} - 3]) &- 1] \\ & + & w_{3} [\bar{I}_{1} - 3]^{2} &+& w_{4} [\exp (w_{4}^{*} [\bar{I}_{1} - 3]^{2} ) &- 1] \end{array}\end{aligned} $$

and the invariant $\bar{I}_2,$

20 $$\begin{aligned} \begin{array}{lclcll} \psi _{{\bar{I}_{2} }} &=& w_{5} [\bar{I}_{2} - 3] &+& w_{6} [\exp (w_{6}^{*} [\bar{I}_{2} - 3]) &- 1] \\ & +& w_{7} [\bar{I}_{2} - 3]^{2} &+& w_{8} [\exp (w_{8}^{*} [\bar{I}_{2} - 3]^{2} ) &- 1]. \end{array} \end{aligned} $$

In addition, we introduce the new the function $\psi _{J}$ guided by previous work [28], to ensure polyconvexity when *J ≠ 1* [34], while satisfying the zero stress condition (13) and the limit conditions (14),

21 $$\begin{aligned} \begin{array}{lclll} \psi _{J} &= & w_{9} &[J^{{w_{9}^{*} }} - w_{9}^{*} \ln (J) &- 1] \\ & + &w_{{10}} &[\exp (w_{{10}}^{*} (\ln (J))^{2} ) &- 1]. \end{array} \end{aligned} $$

Both terms have been previously featured in constitutive models for rubber-like materials [35–37].

#### Mixed-invarint terms.

Each term in Eqs. (19), (20), (21) only depends on a single invariant, $\{\bar{I_1}, \bar{I_2}, J\}$. However, for many foams, the volumetric and deviatoric responses are inherently coupled. For example, foams are often stiffer in tension than in compression due to their porous microstructure. We incorporate this behavior through the following mixed-invariant terms,

22 $$\begin{aligned} \begin{array}{lcl} \psi _{\bar{I}_1, J} & =& w_{11}\,J^{w_{11}^*} \, [\bar{I_1} - 3] \\ \psi _{\bar{I}_2, J} & =& w_{12}\,J^{w_{12}^*} \, [\bar{I_2} - 3] \,. \end{array} \end{aligned}$$

Both terms take larger values in tension than in compression for positive exponents, ${w_{11}^*},{w_{12}^*} > 0,$ and they satisfy the constraint condition (10). However, both terms may violate the polyconvexity condition. In particular, for $w_{11}^* > \frac{2}{3}$ and for all values of $w_{12}^*,$ the strain energies $\psi _{\bar{I}_1, J}$ and $\psi _{\bar{I}_2, J}$ are not polyconvex or even rank-one convex.

#### Principal-stretch terms.

To address the challenge of polyconvexity of the mixed-invariant terms, while still allowing for a notable tension-compression asymmetry, we consult the classical Ogden-Hill foam model [38], with $\psi _{\lambda _i,J} = \sum _{i=1}^{n} {2\,\mu _i}$ $[\lambda _1^{\alpha _i} + \lambda _2^{\alpha _i} + \lambda _3^{\alpha _i} - 3 + [\, J^{-\alpha _i \, \beta _i} -1 ]/{\beta _i} ]/{\alpha _i^2}.$ For the special case with $\beta _i \rightarrow 0,$ we can express this model exclusively in terms of the three principal stretches $\{ \lambda _1,$ $\lambda _2,$ $\lambda _3\}$ as outlined in Sect. 2.4, $\psi _{\lambda _i} = \sum _{i=1}^n {\mu _i}/{\alpha _i^2} \sum _{j=1}^3 [ \lambda _j^{\alpha _i} - \alpha _i \ln (\lambda _j) - 1].$ Here we consider the first two terms of this series, *n = 2,* and obtain the following principal-stretch terms,

23 $$\begin{aligned} \begin{array}{lcl} \psi _{\lambda _i} &=& w_{13} \sum _{j=1}^3 [\,\lambda _j^{w_{13}^*} \!- w_{13}^* \, \ln (\lambda _j) -1\,] \\ & +& w_{14} \sum _{j=1}^3 [\,\lambda _j^{w_{14}^*} \!- w_{14}^* \, \ln (\lambda _j) -1], \end{array} \end{aligned}$$

which we include into the principal-stretch based part of our neural network [26, 39].

#### Constitutive neural network.

**Fig. 3:**
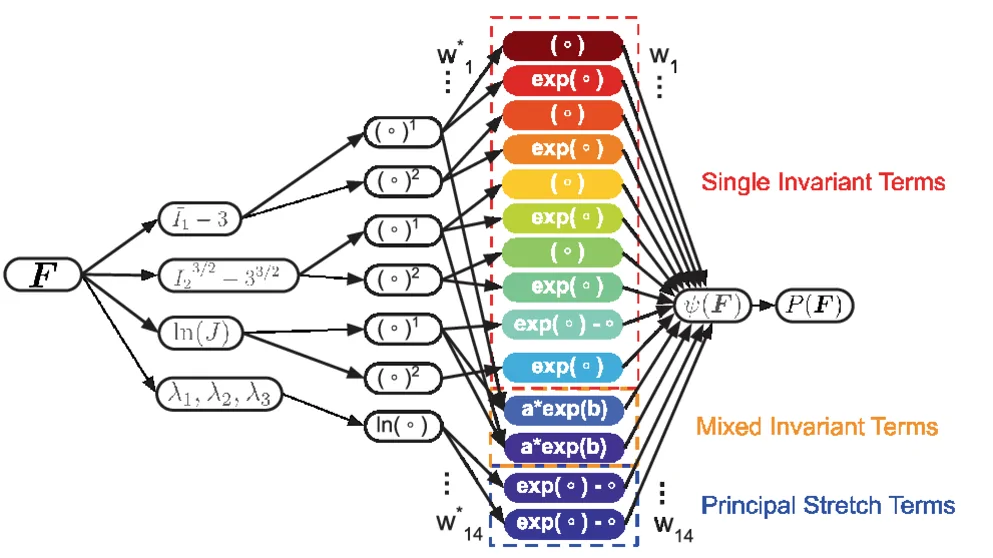
**Constitutive neural network for ultra-low density elastomeric foams.** The network takes sets of three invariants $\bar{I}_1, \bar{I}_2, J$ or three principal stretches $\lambda _1, \lambda _2, \lambda _3$ as input and learns a free energy function *ψ * from which we derive the stress. The free energy function is made up of 14 terms, four first- and four second-invariant terms, $\psi _{\bar{I}_1}$ and $\psi _{\bar{I}_2},$ two third-invariant terms, $\psi _{J},$ two mixed-invariant terms, $\psi _{\bar{I}_1,J}$ and $\psi _{\bar{I}_2,J},$ and two principal-stretch terms, $\psi _{\lambda _i}.$ During training, we selectively activate different combinations of terms to discover the best model for two ultra-low density elastomeric foams

Figure 3 illustrates the complete network architecture for the free energy function (18) as a sum of the 14 individual terms from Eqs. (19) to (23), four first- and four second-invariant terms, $\psi _{\bar{I}_1}$ and $\psi _{\bar{I}_2},$ two third-invariant terms, $\psi _{J},$ two mixed-invariant terms, $\psi _{\bar{I}_1,J}$ and $\psi _{\bar{I}_2,J},$ and two principal-stretch terms, $\psi _{\lambda _i}.$ During automated model discovery, we include different subsets of these 14 terms into network training, and compare the goodness of fit of the discovered models. Some of these models will include only polyconvex terms, but we will also discover and discuss models, which feature non-polyconvex terms.

#### Polyconvexity.

A common requirement is to restrict the free energy function *ψ * to be polyconvex, as this ensures the existence of at least one local minimum [40]. Much research has focused on identifying polyconvex strain energy functions, and finding conditions under which particular strain energy functions are polyconvex [41]. However, microstructural materials such as foams might not always be well-represented by polyconvex functions [42–44], and recent work suggests that global polyconvexity may not be the best requirement to ensure a physically reasonable behavior [45]. In the network architecture in Fig. 3, the single invariant terms, $\psi _{\bar{I}_1}, \psi _{\bar{I}_2}, \psi _{J},$ and the principal-stretch terms, $\psi _{\lambda _i}$ are polyconvex by design, provided that all weight $w_i^*$ and $w_i$ are non-negative. Yet, the mixed invariant terms $\psi _{\bar{I}_1,J}, \psi _{\bar{I}_2,J}$ are not necessarily polyconvex, even for non-negative weights $w_i$ and $w_i^*,$ and we may have to consult alternative solution strategies tailored to non-convex multi-well potentials [46].

### Loss function

To train the constitutive neural network in Fig. 3, we minimize a loss function that consists of a weighted squared error between the model stresses $P(\lambda _i)$ and the experimental stresses $P_i,$ and add an $\textsf {L}_{0.5}$ regularization term that promotes sparsity of the model [47],

24 $$\begin{aligned} \begin{array}{lcll} \textsf {L} & = &\sum _{i=1}^{n_{\text {ten}}} & [ [\, P_{11}(\lambda _i) - P_{11,i} \,]^2 + P_{22}(\lambda _i)^2] / \, (P_{11,i}^{\text {max}})^2\\ & =& \sum _{i=1}^{n_{\text {com}}} &[ [\, P_{11}(\lambda _i) - P_{11,i} \,]^2 + P_{22}(\lambda _i)^2] / \, (P_{11,i}^{\text {min }})^2\\ & + &\sum _{i=1}^{n_{\text {shr}}} &[ [\, P_{12}(\gamma _i) - P_{12,i} \,]^2 / \,(P_{12,i}^{\text {max}})^2 + \sum _{i=1}^{n_{w}} \alpha \, |\,w_i \,|^{0.5} \end{array}\end{aligned}$$

The parameter *α * in the regularization term controls sparse regression and balances the flexibility and accuracy of machine learning models with the familiarity and interpretability of classical constitutive models [48]. First, we train without the regularization term by setting the regularization parameter to zero, *α = 0.* Then, we initialize the network with the learned weights, and systematically vary the regularization parameter *α * [48]. We select the model that has the fewest terms without significantly sacrificing performance relative to the non-regularized model.

## Results

### Mechanical testing

**Fig. 4:**
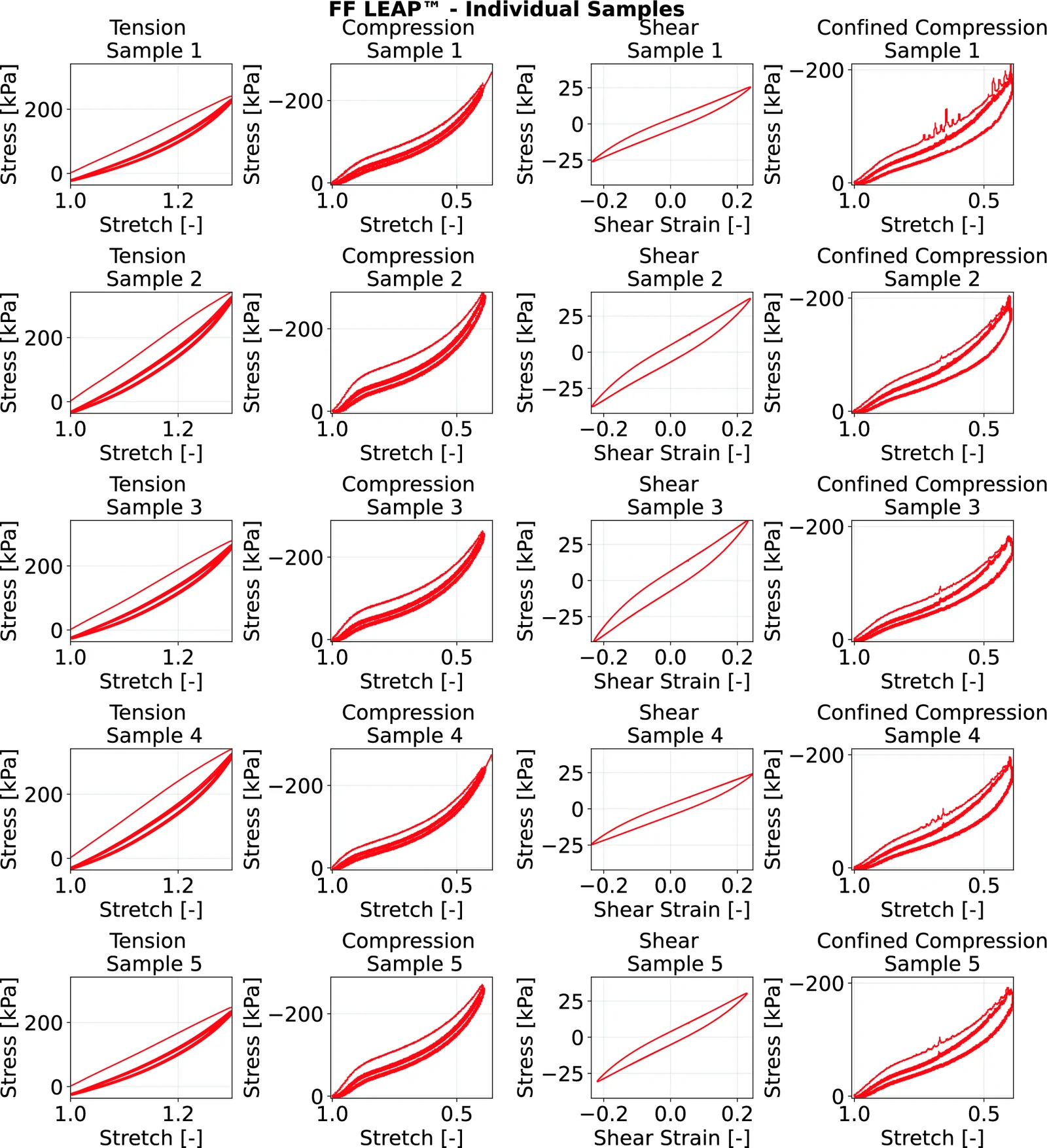
**FF LEAP**$^\mathsf{{TM}}$ **raw data from uniaxial tension, unconfined compression, simple shear, and confined compression experiments.** Piola stress vs stretch or shear strain measurements for all FF LEAP$^\mathsf{{TM}}$ samples. Columns correspond to the experimental setups, tension, compression, shear, and confined compression; rows corresponds to the different samples

**Fig. 5:**
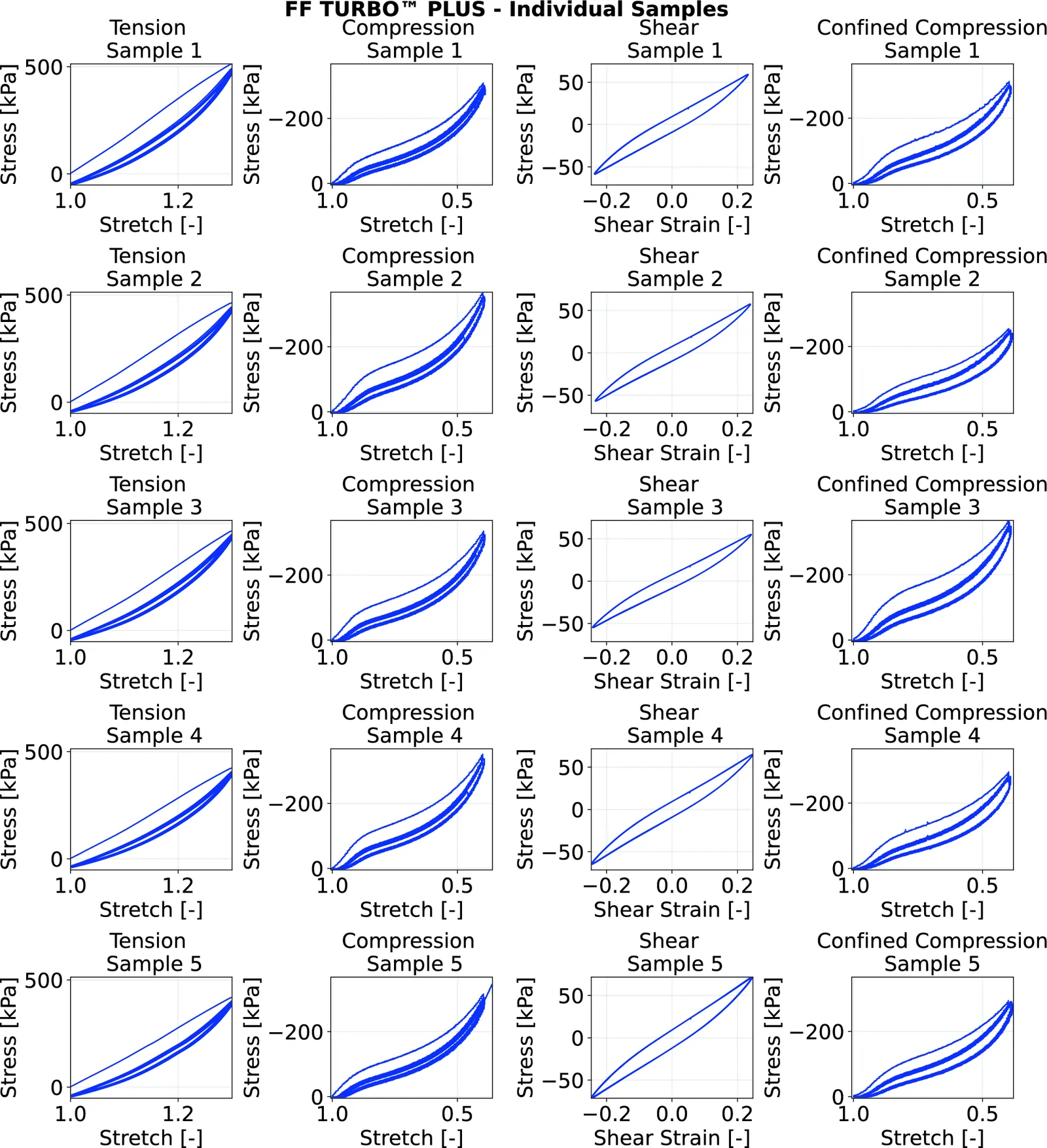
**FF TURBO**$^\mathsf{{TM}}$ **PLUS** **raw data from tension, compression, shear, confined compression experiments.** Piola stress vs stretch or shear strain measurements for all FF TURBO$^\mathsf{{TM}}$ PLUS samples. Columns correspond to the experimental setups, tension, compression, shear, and confined compression; rows corresponds to the different samples

Figures 4 and 5 summarize the raw data from the uniaxial tension, unconfined compression, simple shear, and confined compression experiments of the FF LEAP$^\mathsf{{TM}}$ and FF TURBO$^\mathsf{{TM}}$ PLUS foams.

For all experiments and for both foams, the shapes of the stress-stretch curves appear qualitatively similar across all samples, although the magnitude of the stresses varies considerably between the different tests.

#### Uniaxial tension and compression.

For the uniaxial tension and compression experiments, we see that, except for the first loading curve, all loading and unloading curves follow similar paths. The gap between the unloading and reloading curves is minor, which suggests that there is minimal inelastic dissipation; however, the gap between the first loading curve and all subsequent loading curves is clearly noticeble, which suggests a significant Mullins-type stress softening [49], an initial conditioning effect with minimal subsequent dissipation. To disregard this effect, we exclude the first loading and unloading curves from our analysis as described in Sect. 2.3.

#### Simple shear.

For the simple shear experiments, the gap between the loading and unloading curves is significant, which suggests the presence of notable inelastic dissipation. The mean of the loading and unloading curves is approximately linear, which suggests that in the tested range, the elastic stress response remains fairly linear. Specifically, the coefficient of determination between the shear stress and the shear strain is $R^2 = 0.999$ for both FF LEAP$^\mathsf{{TM}}$ and FF TURBO$^\mathsf{{TM}}$ PLUS.

**Table 1:**
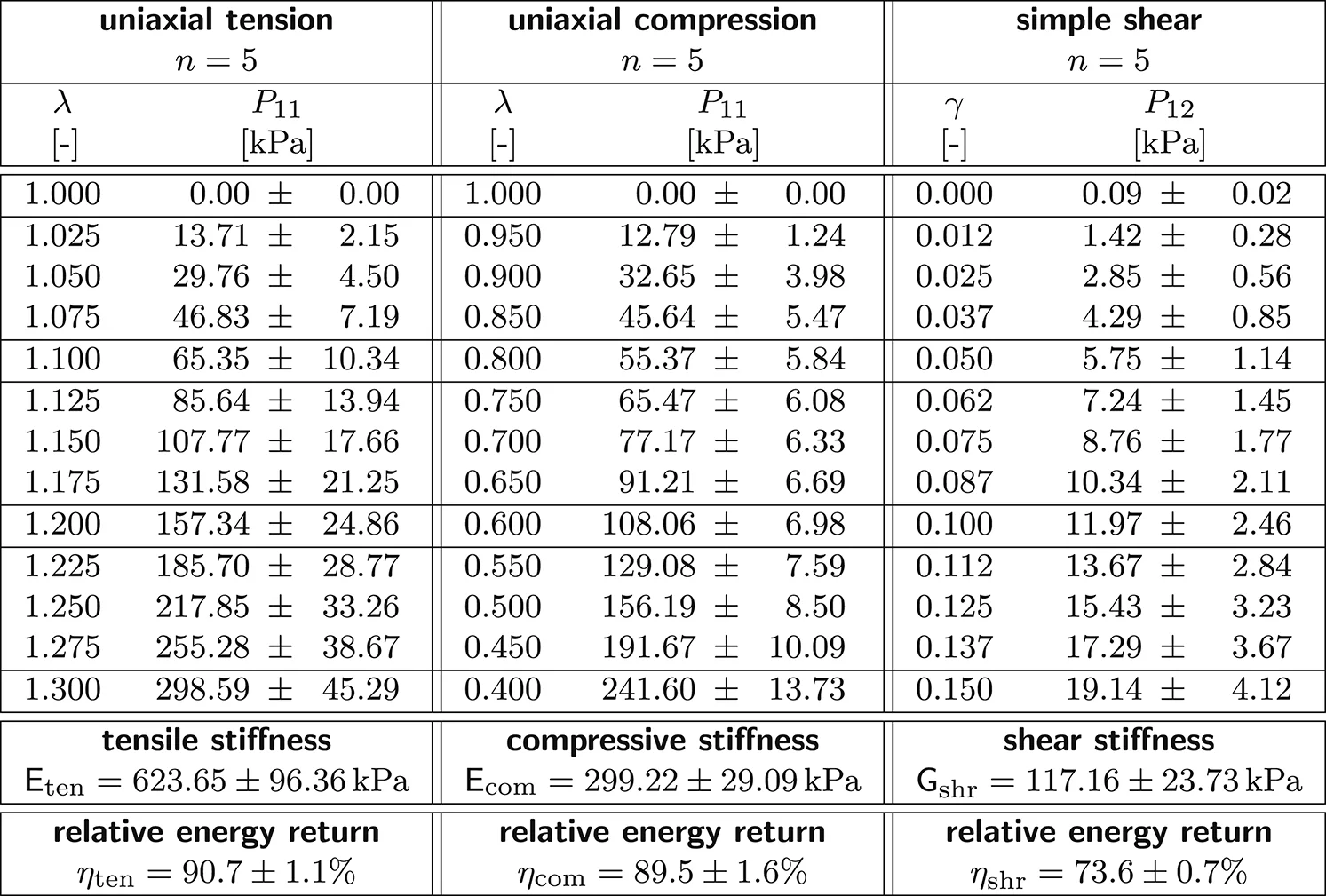
FF LEAP$^\mathsf{{TM}}$ data from tension, compression, shear experiments

Recorded stretch-stress pairs $\{ \lambda, P_{11} \}$ and $\{ \gamma, P_{12}\}$, linear elastic stiffnesses $\textsf {E}$, and relative energy return *η * for the FF LEAP$^\mathsf{{TM}}$ foam. The first two columns represent uniaxial tension, the middle two columns uniaxial compression, and the last two columns simple shear. Means and standard deviations are reported across *n=5* samples

**Table 2:**
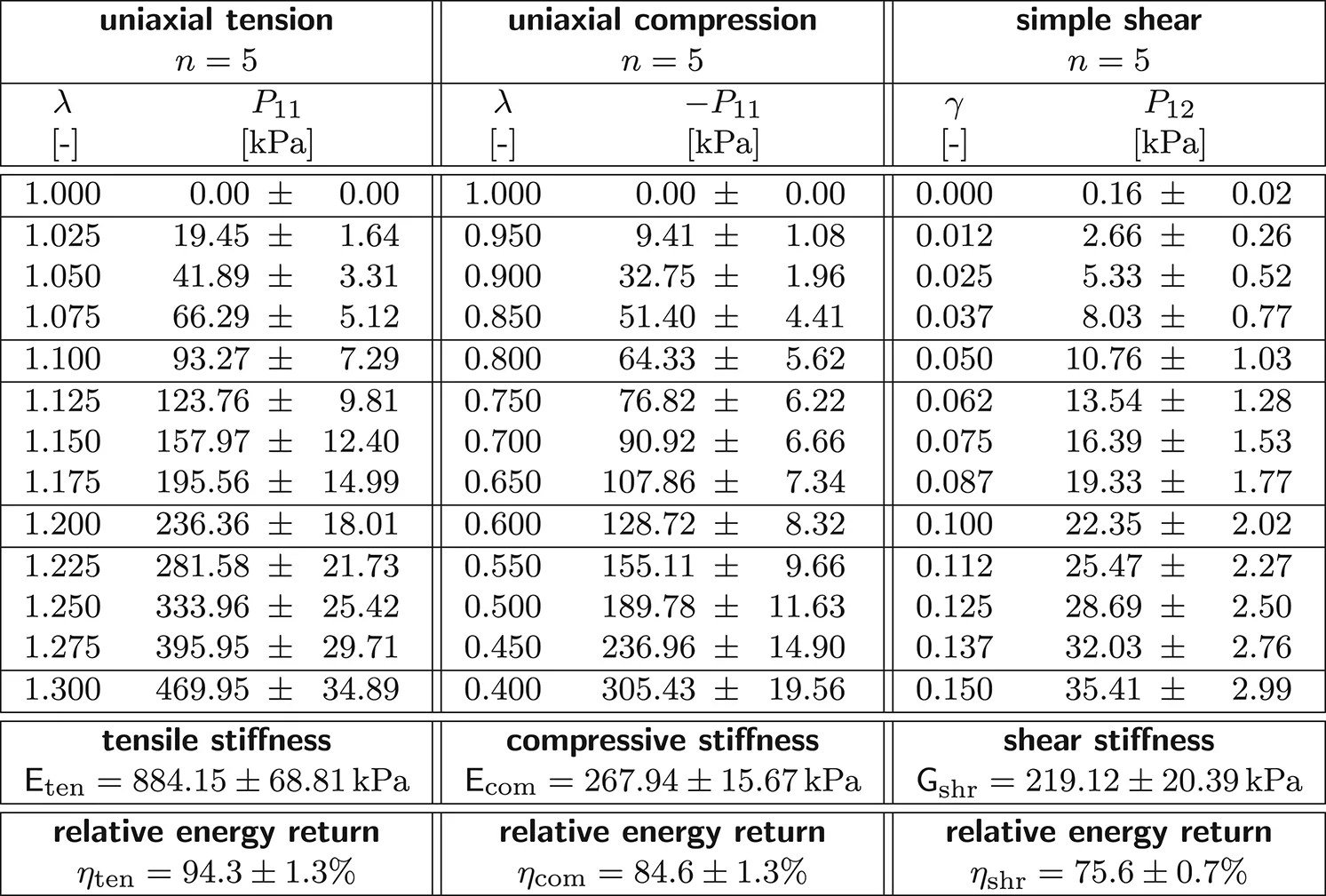
FF TURBO$^\mathsf{{TM}}$ PLUS data from tension, compression, shear experiments

Recorded stretch-stress pairs $\{ \lambda, P_{11} \}$ and $\{ \gamma, P_{12}\}$, linear elastic stiffnesses $\textsf {E}$, and relative energy return *η * for the FF TURBO$^\mathsf{{TM}}$ PLUS foam. The first two columns represent uniaxial tension, the middle two columns uniaxial compression, and the last two columns simple shear. Means and standard deviations are reported across *n=5* samples

**Fig. 6:**
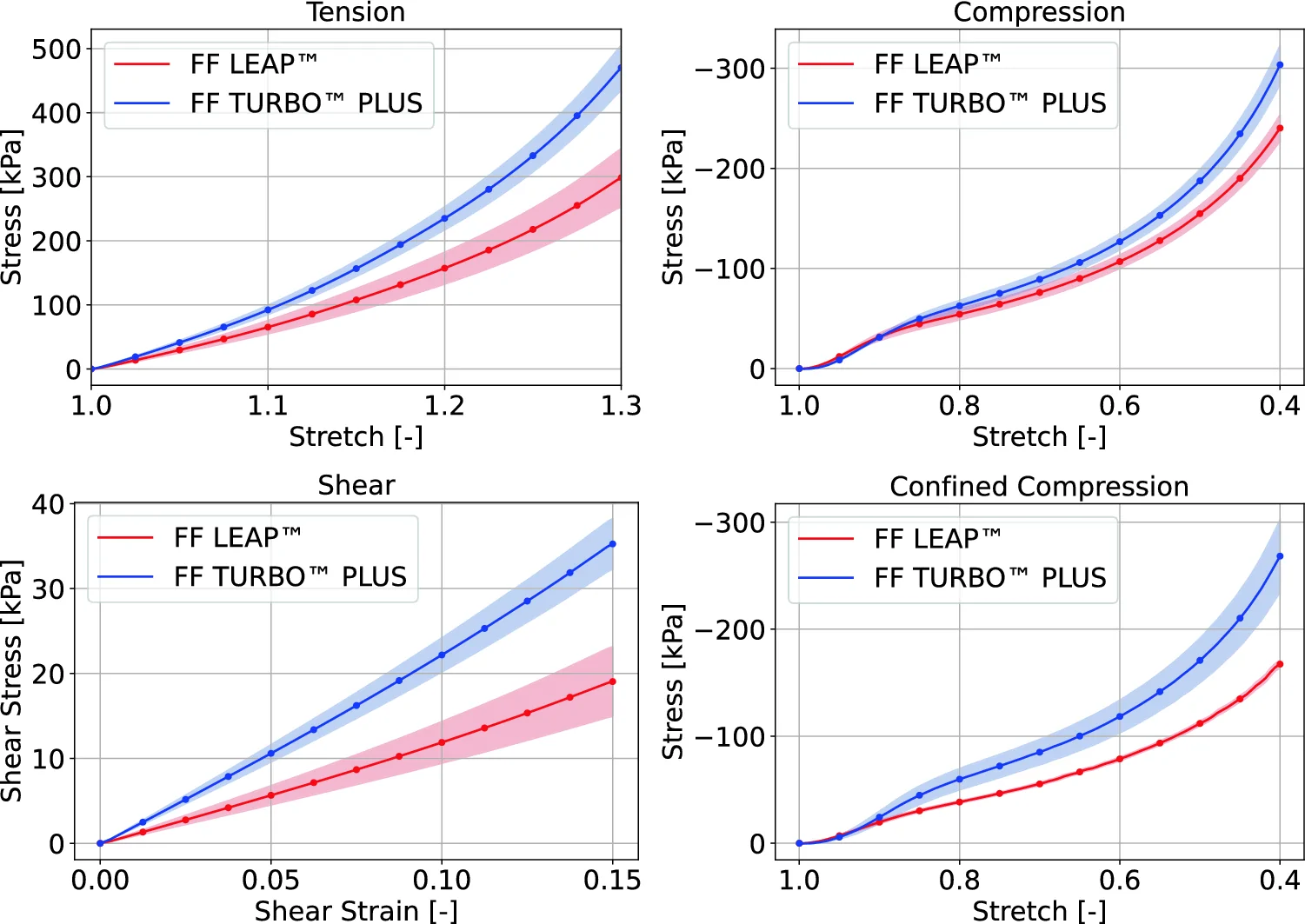
**FF LEAP**$^\mathsf{{TM}}$ **and** **FF TURBO**$^\mathsf{{TM}}$ **PLUS** **data from tension, compression, shear, and confined compression experiments.** Recorded stretch-stress curves $\{ \lambda, P_{11} \}$ and $\{ \gamma, P_{12}\},$ for the FF LEAP$^\mathsf{{TM}}$ foam, shown in red, and the FF TURBO$^\mathsf{{TM}}$ PLUS foam, shown in blue. Dots highlight the discrete values from Tables 1 and 2, lines represent the means, and shaded areas the standard deviations across *n=5* samples

#### Unconfined and confined compression.

For the confined compression experiments, the stress is comparable to the unconfined compression experiment, but slightly lower. We cannot explain this observation with a standard constitutive model, as constraining the transverse stretch should only increase the strain energy compared to unconfined compression. The observed differences may be caused by an earlier onset of microstructural instabilities or could reflect experimental artifacts, imperfect confinement, or frictional effects. The stress drop when switching from loading to unloading during confined compression in Figs. 4 and 5 would support the hypothesis of frictional effects. Overall, we conclude that the transverse stretch in the unconfined and confined compression experiments is similar and equal to, or at least very close to one. Taken together, the similarity of the confined and unconfined compression experiments is consistent with a small effective Poisson’s ratio in compression.

**Table 3:**
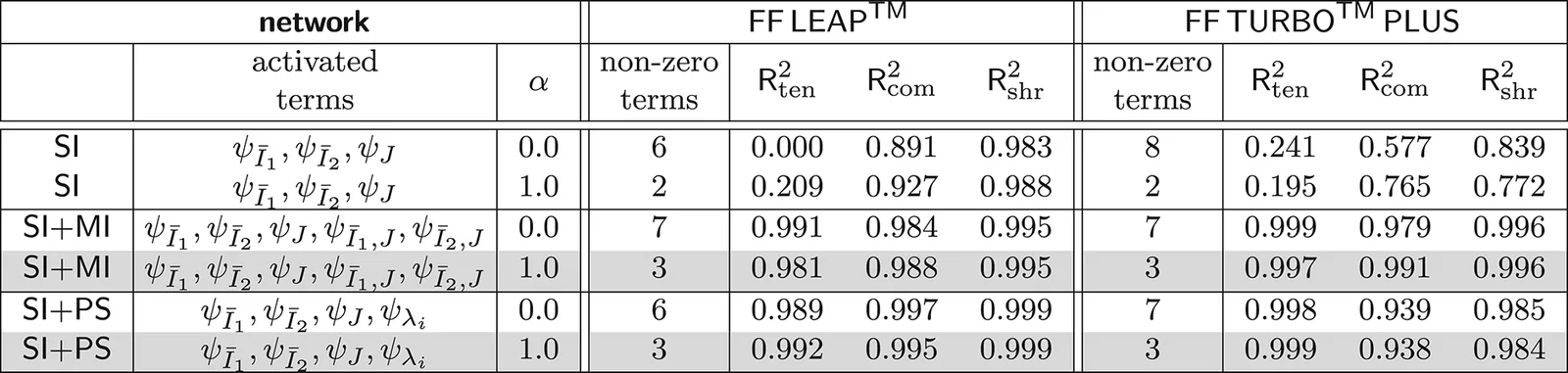
Performance of discovered models for FF LEAP$^\mathsf{{TM}}$ and FF TURBO$^\mathsf{{TM}}$ PLUS

**Fig. 7:**
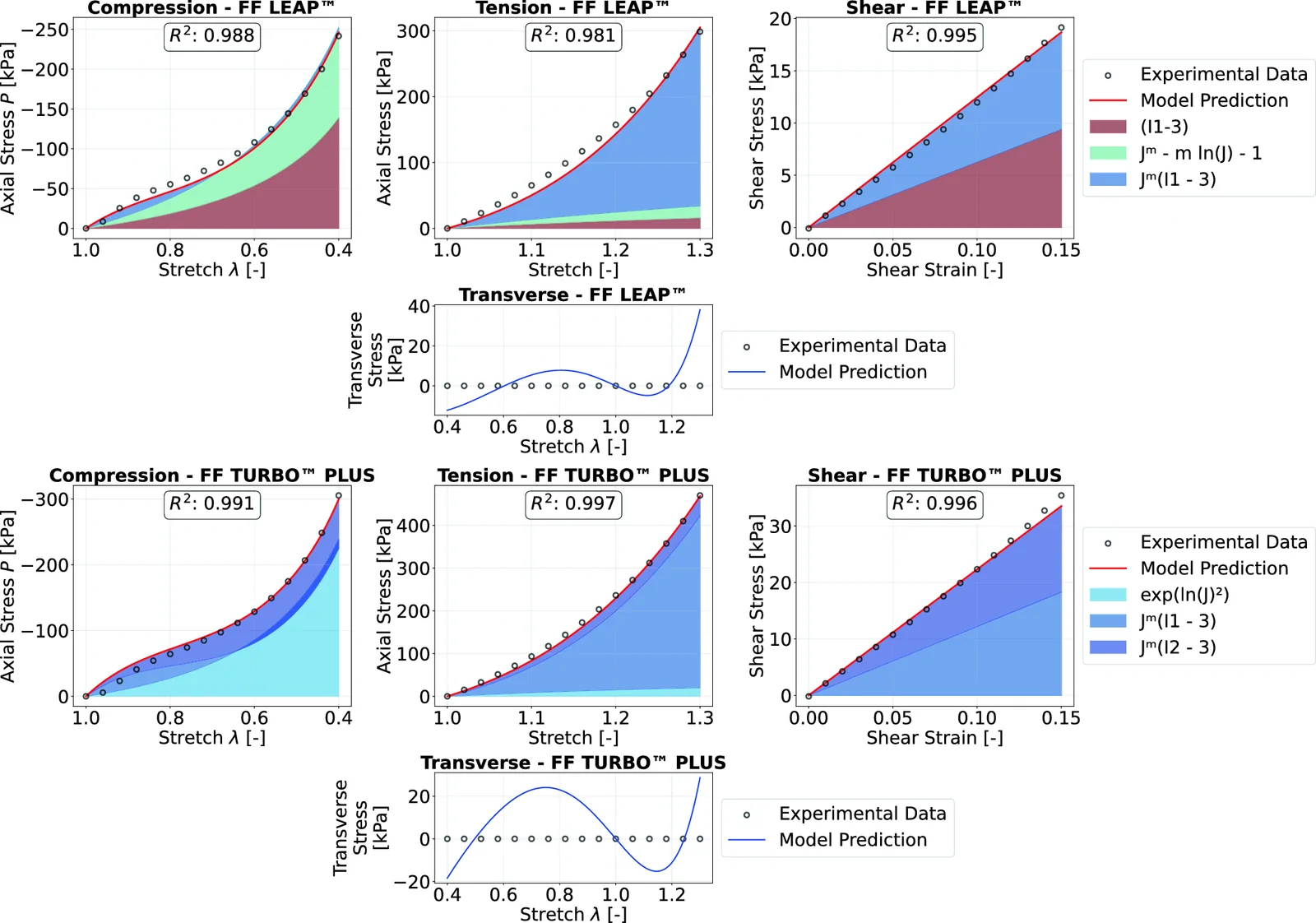
**Discovered model with single-invariant and mixed-invariant terms for** **FF LEAP**$^\mathsf{{TM}}$ **and** **FF TURBO**$^\mathsf{{TM}}$ **PLUS**. Model prediction of constitutive neural network trained with the single-invariant and mixed-invariant terms and $\textsf {L}_{0.5}$ regularization with a regularization parameter *α = 1.0.* For tension, compression, and shear, the contributions from each term are shown in a different color and the ${\textsf {R}}^2$ value between model and data is labeled in each plot. Note that the mixed invariant terms may have a negative stress contribution in compression

**Fig. 8:**
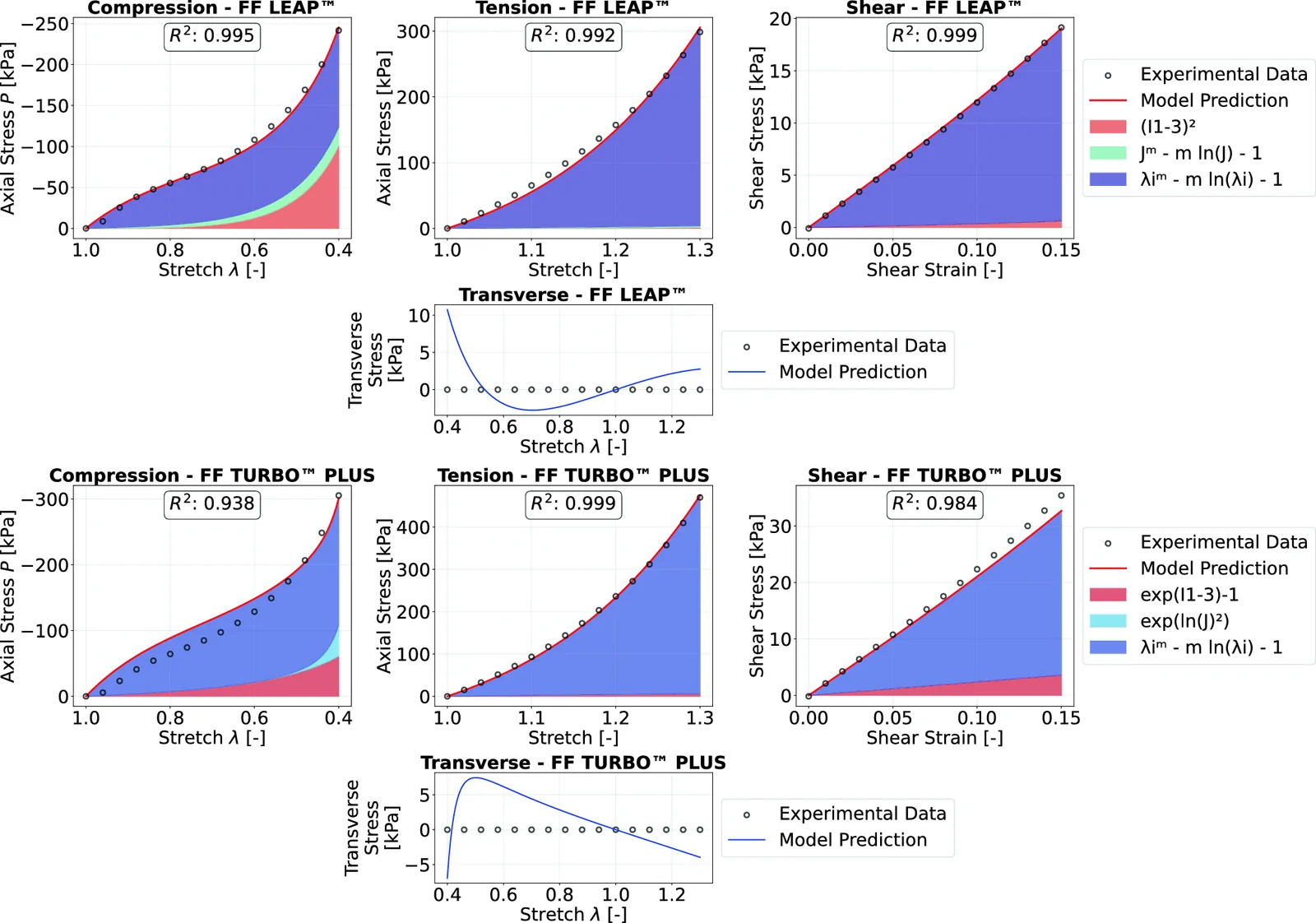
**Discovered model with single-invariant and principal-stretch terms for** **FF LEAP**$^\mathsf{{TM}}$ **and** **FF TURBO**$^\mathsf{{TM}}$ **PLUS**. Model prediction of constitutive neural network trained with the single-invariant and principal-stretch terms and $\textsf {L}_{0.5}$ regularization with a regularization parameter *α = 1.0.* For tension, compression, and shear, the contributions from each term are shown in a different color and the ${\textsf {R}}^2$ value between model and data is labeled in each plot

Activated terms, regularization parameter *α *, number of non-zero terms, and goodness of fit ${\textsf {R}}^2$ in tension, compression, and shear. In each row, we activate a subset of the terms of the network in Fig. 3, with SI single-invariant, MI mixed-invariant terms, and PS principal-stretch terms. We apply either no regularization, with *α =0.0*, or $\textsf {L}_{0.5}$ regularization with *α =1.0*. The gray rows highlight the models in Figs. 7 and 8

### Data analysis

Tables 1 and 2 summarize the stretch-stress pairs, the linear elastic stiffness, and the relative energy return for both foams in tension, compression, and shear, averaged across all *n=5* tests. Figure 6 illustrates the results for both foams in a side-by-side comparison.

#### Tension-compression asymmetry.

For the FF LEAP$^\mathsf{{TM}},$ the linear tensile, compressive, and shear stiffnesses of ${\textsf {E}}_{\text {ten}}=623\pm 96$ kPa and ${\textsf {E}}_{\text {com}}=299\pm 29$ kPa and ${\textsf {G}}_{\text {shr}}=117\pm 24$ kPa clearly highlight a strong tension-compression asymmetry, with a more than twice-as-high resistance to tension compared to compression, and a low resistance to shear, consistent with low-density elastomeric foams. For the FF TURBO$^\mathsf{{TM}}$ PLUS, the linear tensile, compressive, and shear stiffnesses of ${\textsf {E}}_{\text {ten}}=884\pm 69$ kPa and ${\textsf {E}}_{\text {com}}=268\pm 16$ kPa and ${\textsf {G}}_{\text {shr}}=219\pm 20$ kPa show an even more pronounced tension-compression asymmetry, but display a markably higher resistance to shear. The relative energy return mimics these trends: It is compatible for the FF LEAP$^\mathsf{{TM}}$ and FF TURBO$^\mathsf{{TM}}$ PLUS, highest in tension with 90.7±1.1% and 94.3±1.3%, followed by compression with 89.5±1.6% and 84.6±1.3%, and lowest in shear with 73.6±0.9% and 75.6±0.7%.

#### FF TURBO$^\mathsf{{TM}}$ PLUS is stiffer than FF LEAP$^\mathsf{{TM}}$.

Compared with the FF LEAP$^\mathsf{{TM}}$, in the linear regime, the FF TURBO$^\mathsf{{TM}}$ PLUS exhibits a similar compressive stiffness of −10.4% but a significantly larger *(p=0.0017)* tensile stiffness of +41.9%, and a substantially larger *(p=0.00007)* shear stiffness of +87.2%, which indicates a markedly greater resistance to lateral deformation at a comparable level of vertical support. This trend continues into the non-linear regime. At our maximum relative deformations of 30% in tension, 60% in compression, and 15% in shear, FF TURBO$^\mathsf{{TM}}$ PLUS exhibits significantly higher stresses than FF LEAP$^\mathsf{{TM}}$ in tension *(p = 0.033),* compression *(p = 0.001),* and shear *(p = 0.0003),* with the largest difference in shear: Its tensile and compressive stresses are about 25% larger, while its shear stresses are almost twice as large.

### Automated model discovery

Next, we discover models for both foams using the constitutive neural network from Fig. 3. We train on tension, compression, and shear data for 15,000 epochs with a batch size of 64 and a learning rate of 0.01; where, for the first 5,000 epochs, we set the regularization parameter *α * to zero. We the vary the network architecture to include all ten single-invariant terms and either no other terms, or the two mixed-invariant terms, or the two principal-stretch terms. For each architecture, we set the regularization parameter *α * to either 0.0 or 1.0, as our preliminary parameter study has shown that this reduces the number of terms without sacrificing the goodness of fit. Table 3 summarizes the results from training the network for all six cases.

#### Performance of discovered models for FF LEAP$^\mathsf{{TM}}$ and FF TURBO$^\mathsf{{TM}}$ PLUS

The network performs poorly when using only the single invariant terms, especially in tension. However, when adding either the mixed-invariant terms or the principal-stretch terms, the goodness of fit improves drastically. Table 3 confirms that adding regularization to either of these two models significantly reduces the number of terms, from six or seven down to three, and has a minimal impact on the overall goodness of fit. We focus on these two models with a regularization parameter of *α = 1.0,* which provides an excellent goodness of fit while remaining sparse and interpretable. The discovered single-invariant-mixed-invariant model for both foams is

$$\begin{aligned}\begin{array}{lcl} \psi ^{\text {leap}} &=& 26.9 \text{ kPa } \hspace{0.9cm}[\bar{I}_1 - 3] \\ &+& 70.3 \text{ kPa }\,\,\, [J^{1.06} - 1.06 \ln (J) - 1] \\ &+& 79.0 \mathrm{kPa}\,\,\,\,\,J^{4.86} \, [\bar{I}_1 - 3]\\\\ \psi ^{\text {turbo}} &=& 936 \mathrm{kPa}\,\,\,\,[\exp (0.0615 \ln (J)^2) - 1] \\ &+& 139 \text{ kPa }\,\,J^{4.36} \, [\bar{I}_1 - 3] \\ & + &19.8 \mathrm{kPa}\,\,\, J^{1.47} \, [\bar{I}_2 - 3], \end{array}\end{aligned} $$

and the discovered single-invariant-principal-stretch model for both foam is

$$\begin{aligned} \begin{array}{lcl} \psi ^{\text {leap}} &=& 9.93 \text{ kPa }\,\,\,\,[\bar{I}_1 - 3]^2 \\ &+& 59.0 \text{ kPa}\,\,\,\,\, [J^{0.481} - 0.481 \ln (J) - 1] \\ &+& 0.286 \text{ kPa } \sum _{i=1}^3 [\lambda _i^{8.40} - 8.40 \ln (\lambda _i) - 1]\\\\ \psi ^{\text {turbo}}& =&73.9 \text{ kPa}\,\,\,\,\,\,\,\,\,\exp (0.147 (\bar{I}_1 - 3)) - 1] \\ &+& 0.00592 \text{ kPa } [\,\exp (6.64 \ln (J)^2) - 1] \\ &+& 0.365 \text{ kPa}\,\,\,\,\,\,\,\sum _{i=1}^3 [\lambda _i^{8.33} - 8.33 \ln (\lambda _i) - 1]. \end{array} \end{aligned} $$

Figures 7 and 8 show the predicted stress for these two models with the contributions from the three individual terms highlighted in different colors.

## Discussion

We successfully quantified ultra-low-density elastomeric midsole foams in uniaxial tension, unconfined and confined compression, and simple shear using a consistent experimental protocol that resulted in robust and repeatable measurements with low variability across samples. These data enabled the discovery of sparse, interpretable constitutive models that capture the essential mechanical characteristics of each foam across all loading modes.

### Ultra-low-density elastomeric foams display an unconventional, unique mechanical signature.

First and foremost, our experimental tests of both foams reveal the characteristic features of ultra-low-density elastomeric polymeric foams: a pronounced tension–compression asymmetry and a relatively low shear resistance [10]. The close agreement between the unconfined and confined compression tests of both foams indicates a negligible lateral deformation, consistent with an effective Poisson’s ratio close to zero, $\nu \approx 0$ [50]. The narrow loading–unloading loops in both tension and compression indicate a low hysteresis and an efficient energy return [51], 91% and 94% in tension and 90% and 85% in compression, consistent with the high energy return from 76% to 87% reported in elite-level racing shoes [3]. For both foams, tensile stiffness exceeds compressive stiffness, consistent with bending-dominated cellular architectures, in which compression and shear primarily engage cell wall bending, whereas tension activates cell wall stretching [10]. Both foams provide comparable compressive support, 268 kPa vs. 299 kPa, indicating similar vertical cushioning under body-weight loading. In contrast, the FF TURBO$^\mathsf{{TM}}$ PLUS exhibits a 42% higher tensile stiffness, 884 kPa vs. 624 kPa, and nearly double the shear stiffness of the FF LEAP$^\mathsf{{TM}},$ 219 kPa vs. 117 kPa, which implies that it provides substantially greater resistance to lateral deformation. These differences are consistent with the design philosophy of the Asics Metaspeed series, in which the Edge and Sky variants differ primarily in the vertical arrangement of these two foams relative to the carbon-fiber plate. Interestingly, the newest member of the Asics Metaspeed series, the Ray variant, only uses the FF LEAP$^\mathsf{{TM}}$, both above and below the carbon-fiber plate. As long-distance road running is dominated by sagittal-plane loading, the low compressive stiffness of both foams appears well suited for straight-line running efficiency. However, during sharp cornering or curved-path running, the lower shear stiffness of the FF LEAP$^\mathsf{{TM}}$ could increase injury risk, while the higher shear stiffness of the FF TURBO$^\mathsf{{TM}}$ PLUS provides enhanced lateral support and improved stability, especially in curvy and uneven terrain.

### Single-invariant models perform poorly.

For both foams, the classical network [20] with only single invariant terms, $\psi _{\bar{I}_1},\psi _{\bar{I}_2},\psi _J$, fits the data quite poorly, especially in tension. The $\textsf {R}^2$ values of its $\textsf {L}_{0.5}$ regularized version with a penalty of *α = 1.0* range from $\textsf {R}_{\text {ten}}^2=0.20$ in tension to $\textsf {R}_{\text {com}}^2=0.77$ and $\textsf {R}_{\text {com}}^2=0.93$ in compression, to $\textsf {R}_{\text {shr}}^2=0.77$ and $\textsf {R}_{\text {shr}}^2=0.99$ in shear. This suggests that, without mixed-invariant or principal-stretch terms [21], the network cannot accurately capture the characteristic tension-compression asymmetry of ultra-low density elastomeric foams.

### Single-invariant-mixed-invariant models perform excellently.

For the single-invariant-mixed-invariant model with an $\textsf {L}_{0.5}$ regularization penalty of *α = 1.0,* the discovered models for both foams fit the data accurately with only three terms. Their $\textsf {R}^2$ values range from $\textsf {R}^2=0.99$ to $\textsf {R}^2=1.00$ for both foams across all three experiments. For both foam models, the single-invariant terms account for most of stress in compression, while the mixed-invariant terms account for more of the stress in shear and almost the entire stress in tension. The transverse stress remains close to zero across the entire stretch regime, and does not exceed 40 kPa in magnitude. Thus, we conclude that a constitutive neural network with single invariant terms alone is insufficient [52] to achieve a strong tension-compression asymmetry with a much greater stiffness in tension.

### Single-invariant-pricipal-stretch models perform well, but struggle in compression.

For the single-invariant-pricipal-stretch model with an $\textsf {L}_{0.5}$ regularization penalty of *α = 1.0,* the discovered models for both foams fit the data accurately with only three terms. Their $\textsf {R}^2$ values range from $\textsf {R}^2=0.98$ to $\textsf {R}^2=1.00$ for both foams across all three experiments, except for the compression fit of the FF TURBO$^\mathsf{{TM}}$ PLUS with $\textsf {R}_{\text {com}}^2=0.94.$ For both foam models, a single principal-stretch term accounts for the majority of the stress in tension, compression, and shear. However, the additional single-invariant terms are necessary to achieve the appropriate shape of the compression curve. In particular, the stiffening of the foams at small stretches, which is potentially due to densification of the foam [53], cannot be modeled accurately without these additional single-invariant terms. The transverse stress remains close to zero across the entire stretch regime, and does not exceed 15 kPa in magnitude.

### Limitations.

First, our discovered models are hyperelastic by design and cannot predict the amount of energy dissipated during cyclic loading. While both foams exhibit low relative hysteresis, 9% and 6% in tension and 10% and 15% in compression, we observe notable relative hysteresis of 26% and 24% in shear. To accurately model these effects, we would need to collect additional data with varying loading rates and holding times [34], and use inelastic constitutive neural networks [24] to discover inelastic models that explain rate-dependent phenomena. Second, we train our network based on fast loading rates instead of quasi-static data [54]. While this mimics the intended use of the foams, the discovered models may not generalize to other strain rates or loading conditions. Third, we assume that both foams are isotropic and undergo homogeneous deformations. Complementary brightfield microscopy does not reveal any visible anisotropy and suggests that pore sizes are on the order of 100 *μ *m, at least 40*× * smaller than the minimum sample dimension. However, to truly verify isotropy and homogeneity, we would need to perform additional multiaxial experiments. Multiaxial experiments would also allow us to explore a larger space of deformation modes to complement the limited deformation manifold we can explore with uniaxial tension, compression, and shear experiments alone. Last, our discovered models displays small transverse stresses, which we could address with soft or hard constraints, for example, with input specific neural networks [55].

## Conclusion

By performing a series of mechanical tests and using the data to train constitutive neural networks, we have discovered accurate, interpretable, and physics-informed constitutive models for two ultra-low density elastomeric polymeric foams, the FF LEAP$^\mathsf{{TM}}$ and FF TURBO$^\mathsf{{TM}}$ PLUS, used in the Asics Metaspeed Sky and Edge racing shoes. We tested five samples of each foam in uniaxial tension, unconfined and confined compression, and simple shear tests, at a strain rate of 25%/s, over a stretch range from 0.40 to 1.30 and a shear range from 0.00 to 0.25. The measurements revealed that both foams are highly compressible, exhibit pronounced tension–compression asymmetry, and possess an effective Poisson’s ratio close to zero, consistent with their ultra-low density and high porosity. By integrating the data into constitutive neural networks with sparse regression, we discovered compact, interpretable single-invariant models—supplemented by mixed-invariant or principal-stretch based terms—with $\textsf {R}^2$ values close to one across all loading modes. From a human performance perspective, these models enable finite element and gait-level simulations of carbon-fiber—plated racing shoes and provide a quantitative framework for individualized assessment of running economy, performance outcomes, and injury risks across varying running conditions. From a basic-science perspective, these results demonstrate that ultra-light cellular materials require constitutive descriptions that explicitly account for high compressibility and asymmetric deformation mechanisms beyond conventional single-invariant formulations. More broadly, this work establishes a scalable and interpretable framework for constitutive modeling of highly compressible, ultra-light materials, including polymeric foams.

## Data Availability

Source code, data, and examples are available at https://github.com/LivingMatterLab/CANN.

## References

[1] Fernandes GD, Maldonado V (2024) In memory of Kelvin Kiptum: a reflection on his record-breaking marathon and the future outlook for a sub 2-h race from a drafting perspective. Eur J Appl Physiol 124:2379–2388 10.1007/s00421-024-05458-7 38523228

[2] Herzog W (2022) The secrets to running economy. J Sport Health Sci 11:273–274 10.1016/j.jshs.2022.03.003 PMC9189692 35288330

[3] Hoogkamer W et al (2018) A comparison of the energetic cost of running in marathon racing shoes. Sports Med (Auckland NZ) 48:1009–1019 10.1007/s40279-017-0811-2 PMC5856879 29143929

[4] Whiting CS, Hoogkamer W, Kram R (2022) Metabolic cost of level, uphill, and downhill running in highly cushioned shoes with carbon-fiber plates. J Sport Health Sci 11:303–308 10.1016/j.jshs.2021.10.004 PMC9189710 34740871

[5] Patoz A, Lussiana T, Breine B, Gindre C (2022) The Nike Vaporfly 4%: a game changer to improve performance without biomechanical explanation yet. Footwear Sci 14:147–150

[6] Muniz-Pardos B et al (2021) Recent improvements in marathon run times are likely technological, not physiological. Sports Med 51:371–378 10.1007/s40279-020-01420-7 PMC7805427 33442838

[7] Joubert DP, Jones GP (2022) A comparison of running economy across seven highly cushioned racing shoes. Footwear Sci 14:71–83

[8] Rodrigo-Carranza V et al (2024) Influence of different midsole foam in advanced footwear technology use on running economy and biomechanics in trained runners. Scand J Med Sci Sports 34:e14526 10.1111/sms.14526 37858294

[9] Tenforde A, Hoenig T, Saxena A, Hollander K (2023) Bone stress injuries in runners using carbon fiber plate footwear. Sports Med (Auckland Nz) 53:1499–1505 10.1007/s40279-023-01818-z PMC10356879 36780101

[10] Gibson LJ, Ashby MF (1997) Cellular solids: structure and properties, 2nd edn. Cambridge University Press, Cambridge

[11] Tan T, et al (2025) GaitDynamics: a generative foundation model for analyzing human walking and running. Res Square rs.3.rs–6206222 10.1038/s41551-025-01565-8 PMC12928157 41491893

[12] Zöllner AM, Pok JM, McWalter EJ, Gold GE, Kuhl E (2015) On high heels and short muscles: a multiscale model for sarcomere loss in the gastrocnemius muscle. J Theor Biol 365:301–310 10.1016/j.jtbi.2014.10.036 PMC4262722 25451524

[13] Song Y et al (2025) A systematic review of finite element analysis in running footwear biomechanics: insights for running-related musculoskeletal injuries. J Sports Sci Med 24:370–387 10.52082/jssm.2025.370 PMC12131137 40469859

[14] Hannah I, Harland A, Price D, Schlarb H, Lucas T (2016) Evaluation of a kinematically-driven finite element footstrike model. J Appl Biomech 32:301–305 10.1123/jab.2015-0002 26671721

[15] Yong JR et al (2020) Foot strike pattern during running alters muscle-tendon dynamics of the gastrocnemius and the soleus. Sci Rep 10:5872 10.1038/s41598-020-62464-3 PMC7125118 32245985

[16] Yang Z, et al (2022) Design feature combinations effects of running shoe on plantar pressure during heel landing: a finite element analysis with Taguchi optimization approach. Front Bioeng Biotechnol 10 10.3389/fbioe.2022.959842 PMC9513060 36177186

[17] Treloar LRG (1975) The physics of rubber elasticity, 3rd edn. Clarendon Press, Oxford, United Kingdom

[18] Gibson LJ (2003) Cellular solids. MRS Bull 28:270–274

[19] Aimar C, Orgéas L, Rolland du Roscoat S, Bailly L, Ferré Sentis D (2024) Compression fatigue of elastomeric foams used in midsoles of running shoes. Footwear Sci 16:1–11

[20] Linka K, Kuhl E (2023) A new family of constitutive artificial neural networks towards automated model discovery. Comput Methods Appl Mech Eng 403:115731

[21] Linka K, St Pierre SR, Kuhl E (2023) Automated model discovery for human brain using constitutive artificial neural networks. Acta Biomater 160:134–151 10.1016/j.actbio.2023.01.055 36736643

[22] Linka K, Buganza Tepole A, Holzapfel GA, Kuhl E (2023) Automated model discovery for skin: discovering the best model, data, and experiment. Comput Methods Appl Mech Eng 410:116007

[23] Martonová D, Peirlinck M, Linka K, Holzapfel GA, Kuhl E (2024) Automated model discovery for human cardiac tissue: discovering the best model and parameters. Comput Methods Appl Mech Eng 428:117078

[24] Holthusen H, Lamm L, Brepols T, Reese S, Kuhl E (2024) Theory and implementation of inelastic constitutive artificial neural networks. Comput Methods Appl Mech Eng 428:117063

[25] Holthusen H, Linka K, Kuhl E, Brepols T (2026) A generalized dual potential for inelastic constitutive artificial neural networks: a JAX implementation at finite strains. J Mech Phys Solids 206:106337

[26] St Pierre SR, Linka K, Kuhl E (2023) Principal-stretch-based constitutive neural networks autonomously discover a subclass of Ogden models for human brain tissue. Brain Multiphys 4:100066

[27] St Pierre SR et al (2024) The mechanical and sensory signature of plant-based and animal meat. npj Sci Food 8:94 10.1038/s41538-024-00330-6 PMC11568319 39548076

[28] McCulloch JA, Kuhl E (2024) Automated model discovery for textile structures: the unique mechanical signature of warp knitted fabrics. Acta Biomater 189:461–477 10.1016/j.actbio.2024.09.051 39368719

[29] Peirlinck M, Linka K, Hurtado JA, Holzapfel GA, Kuhl E (2025) Democratizing biomedical simulation through automated model discovery and a universal material subroutine. Comput Mech 75:1703–1723 10.1007/s00466-024-02515-y PMC12130129 40470510

[30] Liang G, Chandrashekhara K (2008) Neural network based constitutive model for elastomeric foams. Eng Struct 30:2002–2011

[31] St Pierre SR, et al (2023) Discovering the mechanics of artificial and real meat. Comput Methods Appl Mech Eng 415:116236

[32] Ogden RW (1972) Large deformation isotropic elasticity—on the correlation of theory and experiment for incompressible rubberlike solids. Proc R Soc A 326:565–584

[33] Valanis K, Landel RF (1967) The strain-energy function of a hyperelastic material in terms of the extension ratios. J Appl Phys 38:2997–3002

[34] Boes B, Simon J-W, Holthusen H, Kuhl E (2026) The mechanics and physics of tofu: understanding hydrated soft solids through feature networks. J Mech Phys Solids 213: 106632

[35] Hencky H (1928) Über die Form des Elastizitatsgesetzes bei ideal elastischen Stoffen. Zeitschrift für Technische Physik 9:215–220

[36] Neff P, Ghiba I-D, Lankeit J (2015) The exponentiated Hencky-logarithmic strain energy. Part I: constitutive issues and rank-one convexity. J Elast 121:143–234

[37] Landauer AK, Li X, Franck C, Henann DL (2019) Experimental characterization and hyperelastic constitutive modeling of open-cell elastomeric foams. J Mech Phys Solids 133:103701

[38] Hill R (1979) Aspects of invariance in solid mechanics. Adv Appl Mech 18:1–75

[39] Buganza Tepole A, Jadoon AA, Rausch M, Fuhg JN (2025) Polyconvex physics-augmented neural network constitutive models in principal stretches. Int J Solids Struct 320:113469

[40] Ball JM (1976) Convexity conditions and existence theorems in nonlinear elasticity. Arch Ration Mech Anal 63:337–403

[41] Hartmann S, Neff P (2003) Polyconvexity of generalized polynomial-type hyperelastic strain energy functions for near-incompressibility. Int J Solids Struct 40:2767–2791

[42] Suchocki C, Jemioło S (2021) Polyconvex hyperelastic modeling of rubberlike materials. J Braz Soc Mech Sci Eng 43:352

[43] Joshi S, Tan J, Hamel CM, Gaitanaros S, Bouklas N (2026) Influence of heterogeneity on the response of architected metamaterials. arXiv:2604.25075

[44] Joshi S et al (2026) Instabilities and phase transitions in architected metamaterials: a gradient-enhanced continuum approach. Comput Methods Appl Mech Eng 452:118719

[45] Wollner MP, Holzapfel GA, Neff P (2026) In search of constitutive conditions in isotropic hyperelasticity: polyconvexity versus true-stress-true-strain monotonicity. J Mech Phys Solids 209:106465

[46] Jones RE, Buganza Tepole A, Fuhg JN (2025) Differentiable neural network representation of multi-well, locally-convex potentials. arXiv:2506.17242

[47] Frank IE, Friedman JH (1993) A statistical view of some chemometrics regression tools. Technometrics 35:109–135

[48] McCulloch JA, St Pierre SR, Linka K, Kuhl E (2024) On sparse regression, Lp-regularization, and automated model discovery. Int J Numer Methods Eng 125:e7481

[49] Mullins L (1969) Softening of rubber by deformation. Rubber Chem Technol 42:339–362

[50] Lakes R (1987) Foam structures with a negative Poisson’s ratio. Science 235:1038–1040 10.1126/science.235.4792.1038 17782252

[51] Gent AN (2012) Engineering with rubber: H ow to design rubber components, 3rd edn. Hanser Publishers, Munich, Germany

[52] Blatz PJ, Ko WL (1962) Application of finite elastic theory to the deformation of rubbery materials. Trans Soc Rheol 6:223–252

[53] Prabhakar P, Feng H, Subramaniyan SP, Doddamani M (2022) Densification mechanics of polymeric syntactic foams. Composites Part B Eng 232:109597

[54] Reed N, Huynh NU, Rosenow B, Manlulu K, Youssef G (2020) Synthesis and characterization of elastomeric polyurea foam. J Appl Polym Sci 137:48839

[55] Jadoon A, Seidl DT, Jones RE, Fuhg JN (2025) Input specific neural networks. arXiv:2503.00268

